# Real-World Utilization of Molnupiravir during the COVID-19 Omicron Surge in Israel

**DOI:** 10.3390/epidemiologia4030031

**Published:** 2023-08-10

**Authors:** Clara Weil, Tobias Bergroth, Anna Eisenberg, Yohance Omar Whiteside, Yoseph Caraco, Lilac Tene, Gabriel Chodick

**Affiliations:** 1 Maccabi Institute for Research and Innovation, Maccabi Healthcare Services, Tel Aviv 68125, Israel; 2 Center for Observational and Real-World Evidence (CORE), MSD, 113 30 Stockholm, Sweden; 3 MSD, Hod Hasharon 45240, Israel; 4 Center for Observational and Real-World Evidence (CORE), Merck & Co., Inc., Rahway, NJ 07065, USA; 5 Clinical Pharmacology Unity, Hadassah-Hebrew University Medical Center, Jerusalem 911200, Israel; 6 School of Public Health, Faculty of Medicine, Tel Aviv University, Ramat Aviv 6997801, Israel

**Keywords:** SARS-CoV-2, COVID-19, molnupiravir

## Abstract

Molnupiravir (MOV) was introduced in Israel in January 2022 during the SARS-CoV-2 Omicron surge for high-risk patients contraindicated for nirmatrelvir/ritonavir. This retrospective cohort study aimed to describe characteristics of patients offered COVID-19 antiviral treatment in Maccabi Healthcare Services (antiviral treatment-eligible cohort; *n* = 5596) between 12 January and 28 February 2022, and the subset of these who were dispensed MOV (MOV-treated cohort; *n* = 1147), as well as outcomes following MOV dispensation. Median (interquartile range) age in the antiviral treatment-eligible and MOV-treated cohorts were 70.5 (61.1, 77.3) and 74.1 (64.3, 81.7) years, respectively. The MOV-treated cohort (male: 53.2%) had high rates of COVID-19 vaccination (91.4%) and comorbidities, including immunosuppression (40.0%) and chronic kidney disease (67.0%; eGFR < 30 mL/min/1.73 m^2^: 28.8%), and most used comedications either contraindicated or with major potential for drug–drug interactions with nirmatrelvir/ritonavir (87.3%). At 28 days post-MOV dispensation, the cumulative incidence (95% CI) of COVID-19-related hospitalization and/or all-cause mortality was 3.6% (2.5%, 4.6%), with similar rates across sexes and age groups (18–64 vs. ≥65 years), and lower rates among recently vaccinated and/or recently SARS-CoV-2-infected patients. These data describe the characteristics and outcomes for MOV-treated patients in Israel, whose clinical characteristics may preclude the use of nirmatrelvir/ritonavir to treat their COVID-19 infection.

## 1. Introduction

Coronavirus disease (COVID-19), caused by severe acute respiratory syndrome coronavirus 2 (SARS-CoV-2), is continuing to challenge healthcare systems with the emergence of new variants, incomplete vaccine coverage, and waning vaccine immunity [1]. Older adults and those with chronic medical conditions have experienced substantially higher risks of hospitalization and mortality from COVID-19 [2,3,4,5]. Although vaccines have significantly reduced the morbidity and mortality associated with COVID-19, [6,7] the virus continues to infect even vaccinated individuals, particularly those in high-risk groups [8].

New treatments against COVID-19 are being developed to complement vaccination programs and potentially decrease the incidence of COVID-19-related hospitalization and mortality. Clinical guidelines recommend antiviral treatment for non-hospitalized adults with symptomatic COVID-19 at risk of disease progression [9,10]. The currently preferred antiviral is the combination of nirmatrelvir and ritonavir [11]. Nirmatrelvir/ritonavir is contraindicated for patients with severe renal and/or hepatic impairment and requires dose adjustment in moderate renal impairment [10]. In addition, ritonavir has numerous drug–drug interactions due to its inhibitory effect on drug metabolism in the liver [10], making nirmatrelvir/ritonavir unsuitable for patients taking certain other medications. MOV, a broad-spectrum ribonucleoside analog inhibitor of SARS-CoV-2, was shown in the MOVe-OUT trial to significantly reduce the risk of COVID-19 hospitalizations or mortality for high-risk patients when given within five days of symptom onset [12]. 

Israel introduced both nirmatrelvir/ritonavir and MOV during the SARS-CoV-2 Omicron surge in January 2022, following emergency use authorization for patients up to 5 days from symptom onset with risk factors for progression to severe disease. MOV was available for high-risk patients for whom nirmatrelvir/ritonavir treatment was not clinically appropriate [10]. Monoclonal antibodies for the treatment of COVID-19 were not used in Israel during the study period, and MOV was the sole treatment option for many high-risk patients.

Data regarding outcomes following MOV treatment in real-world settings are still limited [13,14,15]. Recent studies of MOV-treated patients in Italy [16], Japan [17], and Romania [18] have demonstrated the real-world value of MOV from single-arm studies. Using electronic healthcare data from a large health plan in Israel, this single-arm study aimed to describe the characteristics of patients offered COVID-19 antiviral treatment and the subset of these who were dispensed MOV and to describe outcomes following MOV dispensation overall and by major risk factors. Because MOV was previously shown to significantly reduce the risk of COVID-19 hospitalizations and mortality for high-risk patients [12], and methodological challenges limited the ability to define a suitable comparison group of untreated patients, this study did not seek to assess comparative effectiveness but sought to describe the real-world utilization of MOV using a single-arm study design.

## 2. Materials and Methods

### 2.1. Data Source

A retrospective cohort study was performed using the electronic healthcare data of Maccabi Healthcare Services (MHS), the second largest of Israel’s four state-mandated health plans (payer and provider). MHS covers about a quarter of the population nationwide (>2.6 million members), with longitudinal patient-level data available since 1998. The database includes demographic data and medical histories such as diagnosis codes (International Classification of Diseases, Ninth Revision, Clinical Modification [ICD-9-CM]), laboratory test results, procedures (Current Procedural Terminology codes), and pharmacy data (prescribed and dispensed medications, using a coding system which maps to Anatomical Therapeutic Chemical codes). SARS-CoV-2 laboratory data are captured from PCR and antigen tests performed in MHS and other authorized laboratories in Israel via the Israeli Ministry of Health (MoH). Data on SARS-CoV-2 tests performed outside of these settings were not available for this study.

### 2.2. Study Population

To identify the study population, data were obtained from records of the MHS COVID-19 antiviral treatment center, which was operated by a multi-disciplinary team and was responsible for the delivery of COVID-19 antiviral treatment (nirmatrelvir/ritonavir or MOV) to eligible patients in early 2022. Patients with known risk factors for the severe disease were identified within 24 h of patients’ positive SARS-CoV-2 test results. Each patient’s eligibility for treatment was reviewed by a physician, and patients deemed eligible were contacted by telephone and offered treatment. The patient’s decision to accept (by providing verbal consent) or decline treatment was recorded. For patients who accepted treatment, the process was designed to deliver the medication to their home on the same day. These records were linked (by unique patient identification number) to the treatment dispensation date together with all other data available in the central MHS database. 

In order to describe patient characteristics, the overall study population included an ‘antiviral treatment-eligible cohort’ of non-hospitalized adults (age ≥ 18 years) with a positive SARS-CoV-2 PCR or antigen test result between 12 January and 28 February 2022 who were offered any COVID-19 antiviral treatment (nirmatrelvir/ritonavir or MOV) and had a record of having either accepted or declined treatment and were deemed at high risk for deterioration of COVID-19. For this study, we used a risk score that accounts for comorbidities, prior hospitalization, and COVID-19 vaccination history to exclude patients 50–69 years old with a risk score of <2 points and patients 18–49 years-old with <4 points to ensure that all patients had documented evidence of major risk factors available in the database for this analysis (Supplementary Table S1). 

The MOV-treated cohort, which was the focus of the single-arm outcomes analysis, consisted of the subset of patients from the antiviral treatment-eligible cohort who were dispensed MOV up to 5 days after their SARS-CoV-2 positive test. The index date was defined as the first MOV dispensation, occurring between 17 January (when MOV became available) and 28 February 2022. 

**Figure 1:**
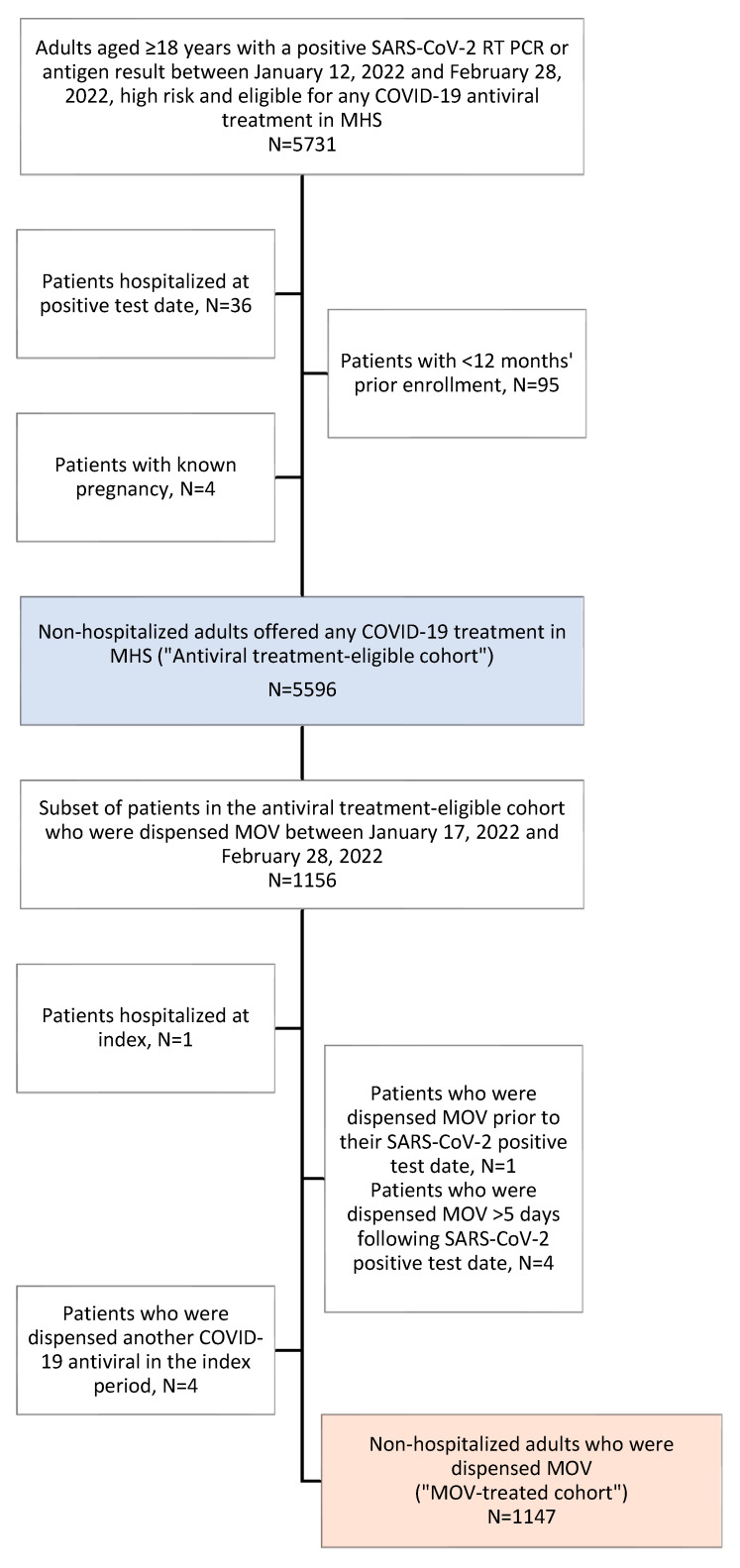
Flowchart for selection of the study population.

All included patients were required to have at least 12 months of continuous health plan enrolment prior to their SARS-CoV-2 positive test and at least 3 months without prior SARS-CoV-2 infection (positive PCR or antigen result). Pregnancy was excluded based on documented diagnosis/procedural codes. In the MOV-treated cohort, patients were excluded if they met any of the following criteria: hospitalization at the index date, more than one purchase date of MOV in the index period, and treatment with nirmatrelvir/ritonavir during the index period (Figure 1).

### 2.3. Variables

#### 2.3.1. Outcomes following MOV Treatment

The primary outcome measures within 28 days post-index were (a) COVID-19-related hospitalization, (b) all-cause hospitalization, (c) all-cause mortality, and (d) a composite outcome of COVID-19-related hospitalization and/or all-cause mortality. These outcomes were defined as follows: (a) COVID-19-related hospitalization was defined as a hospitalization in a COVID-19 ward and/or a hospitalization listed in the Israeli MoH registry of hospitalizations due to COVID-19; (b) all-cause hospitalization included COVID-19-related hospitalizations as well as any overnight stay in any department; (c) all-cause mortality was based on the date of death obtained from the MHS database, which is routinely updated with data from the Israeli national social security insurance system; and (d) a composite outcome of ‘a’ and ‘d’, whereby the outcome date was defined as the date of COVID-19 hospitalization if it occurred within 28 days post-index (irrespective of whether the patient subsequently died in the follow-up period), or the date of death if there was no COVID-19-related hospitalization during follow-up.

In addition, all-cause healthcare resource utilization (HCRU) was described in terms of the frequency of outpatient and inpatient encounters within 28 days post-index. Outpatient encounters were physician office or hospital outpatient visits, specialist visits, virtual/telemedicine meetings, emergency department visits, and after-hour clinic visits. Inpatient encounters were analyzed as the number of hospital and intensive care unit (ICU) admissions and the cumulative lengths of stay (LOS) during the 28-day follow-up period.

#### 2.3.2. Characteristics of Patients in the Antiviral Treatment-Eligible Cohort and MOV-Treated Cohort

Baseline data were obtained to characterize patients in the antiviral treatment-eligible cohort at the time of their SARS-CoV-2 positive test date and to characterize patients in the MOV-treated cohort at index date. The baseline period was 12 months unless otherwise specified. Patients’ COVID-19 vaccination status (with the Pfizer-BioNTech vaccine available at that time to the study population) was recorded as the number of doses, days since the last dose, and vaccination status up to 180 days prior to therapy initiation. Any previous SARS-CoV-2 infection was captured based on positive PCR and antigen test results; infection up to 180 days prior to therapy initiation was reported separately. In the MOV-treated cohort, the number of days from the SARS-CoV-2 positive test date to the MOV index date was also recorded. 

Sociodemographic characteristics obtained were sex, age, residential area, and residential socioeconomic status (SES). SES was based on a score ranked from 1 (lowest) to 10 on an individual’s residence place at the neighborhood level [19,20]. Previous research on COVID-19 in Israel has also used place of residence for classifying the socioeconomic status of individuals [20]. SES was categorized as low (1–4), medium (5–6), and high (7–10). 

Clinical characteristics were BMI, smoking status (ever, never), and the presence of comorbidities. BMI was classified as underweight, normal, overweight, and obese, using BMI kg/m^2^ cutoffs of <18.5, 18.5–24.9, 25–29.9, and ≥30.0, respectively. Comorbidities were identified by existing MHS disease registries for chronic diseases (since 1998) [21,22,23,24], where available, or by ICD-9-CM diagnosis codes (for the past 12 months). Comorbid conditions included conditions associated with an increased risk of severe COVID-19 disease, according to the Centers for Disease Control and Prevention 2021 list, [25] as well as disaccharide/monosaccharide deficiency and malabsorption and pulmonary hypertension. The estimated glomerular filtration rate (eGFR) before/on the index date was reported based on the most recent result up to 6 months pre-index.

Comedications that were either contraindicated to or had the potential for major drug–drug interactions with, nirmatrelvir/ritonavir [26,27,28] (Supplementary Table S2) were identified in the MHS pharmacy database and were defined as ≥1 purchase of medication during the period from 90 days before through 30 days after the MOV index date.

### 2.4. Statistical Analysis

Descriptive statistics were reported. Numbers and percentages were provided for dichotomous and categorical variables. Continuous variables were inspected for normality (Kolmogorov–Smirnov test) and summarized accordingly as mean and standard deviation (SD) or median and inter-quartile range (IQR), with minimum and maximum values. Missing data were presented as a separate category (e.g., BMI category = Missing). In order to investigate the cumulative incidence within 28 days after the MOV index date, as well as the timing of events within the 28-day follow-up period, overall and for specific patient subgroups, Kaplan Meier analysis was used, and Log-rank *p*-value was reported for statistical comparisons between strata for risk major factors (such as age group, vaccination status). Cumulative incidence rates were reported with 95% confidence intervals (CI). All analyses were performed using IBM-SPSS v.28 and R statistical software v.4.0.2.

## 3. Results

### 3.1. Study Population

Among 5731 adults with a positive SARS-CoV-2 RT PCR or antigen result between 12 January 2022 and 28 February 2022, who were defined as high risk and offered any COVID-19 antiviral treatment in MHS, 135 were excluded due to being hospitalized, pregnant, or enrolled in MHS less than 12 months before their SARS-CoV-2 positive test (Figure 1). A total of 5596 patients met the study criteria for inclusion in the antiviral treatment-eligible cohort. Of these, 1147 patients were dispensed MOV up to 5 days after their SARS-CoV-2 positive test and met the criteria for inclusion in the MOV-treated cohort (Figure 1). 

### 3.2. Patient Characteristics

Characteristics of patients in the antiviral treatment-eligible cohort and the subset of patients treated with MOV (MOV-treated cohort) are described in Table 1. 

The MOV-treated cohort included 1147 patients with relatively older age (median = 74.1; IQR = 64.3, 81.7 years) compared to the overall antiviral treatment-eligible cohort, with similar sex distribution (53.2% male). Most patients in the MOV-treated cohort (96.4%) initiated MOV treatment in 0–2 days (vs. 3–5 days) following the positive COVID-19 test result. The MOV-treated cohort was characterized by a high COVID-19 vaccination rate (ever: 91.4%; 180 days prior: 67.5%) and high prevalence of immunosuppression (39.9%) and chronic comorbidities such as hypertension (80.9%), chronic kidney disease (66.7%) and cardiovascular disease (57.0%). A total of 28.8% of patients had a recent eGFR result <30 mL/min/1.73 m^2^. In the MOV-treated cohort, 87.3% were dispensed at least one comedication that was contraindicated to, or had the potential for major drug–drug interactions with, nirmatrelvir/ritonavir, again most frequently cardiovascular (67.5%) and antithrombotic (40.8%) medications (Supplementary Table S3). 

### 3.3. Outcomes following MOV Treatment

**Figure 2:**
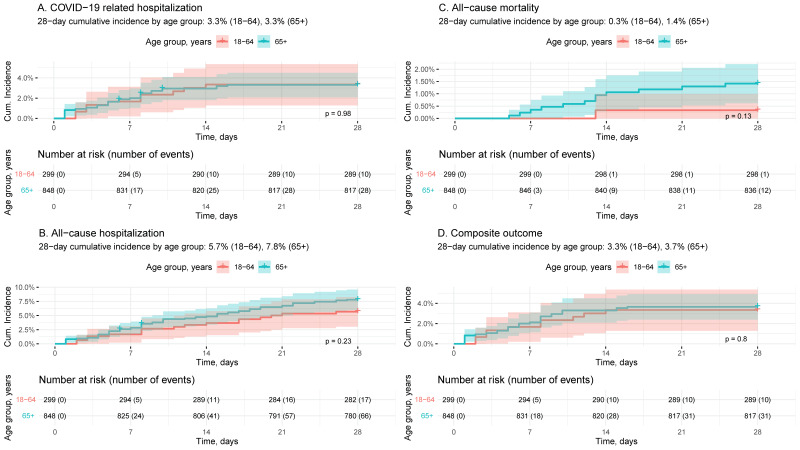
Outcomes following MOV treatment by age group: (**A**) COVID-19-related hospitalization; (**B**) all-cause hospitalization; (**C**) all-cause mortality; and (**D**) a composite outcome of COVID-19-related hospitalization and/or all-cause mortality.

Cumulative incidence rates for each of the following outcomes at 28 days post-index in the MOV-treated cohort are summarized overall and by risk factors in Table 2: A. COVID-19-related hospitalization; B. all-cause hospitalization; C. all-cause mortality; and D. the composite outcome of COVID-19-related hospitalization and/or mortality. These outcomes are depicted by age group (18–64 vs. ≥65 years) in Figure 2.

The cumulative incidence of COVID-19-related hospitalization and/or mortality at 28 days post-MOV dispensation was 3.6% (95% CI: 2.5%, 4.6%), with similar rates across sexes (Supplementary Figure S1) and age groups. Lower rates were seen among recently vaccinated and/or recently SARS-CoV-2-infected patients (Table 2 and Supplementary Figure S2). Excluding underweight (*n* = 10), BMI was not significantly associated with COVID-19-related hospitalization and/or mortality. A total of 13 patients died (12 of whom were ≥65 years of age), corresponding to a cumulative all-cause mortality of 1.1% (95% CI: 0.5%, 1.7%). The incidence of all-cause mortality and all-cause hospitalization was numerically higher in the ≥65 than in the 18–64 age group (Figure 2). 

HCRU following MOV treatment is described in Supplementary Table S4. Almost all patients (98.6%) recorded one or more primary care visits, with a median of 2 visits. About one-third (35.7%) visited a specialist, and 9.4% visited the ER or after-hour urgent care. Half of the MOV-treated patients had at least one telemedicine visit. Among 83 patients (7.2%) who were hospitalized for any cause (for a median stay of 4 days), COVID-19-related hospitalization was recorded in 38 patients (3.3%), with a median stay of 4.5 days. Overall, 12 patients (1.1%) were admitted to the ICU. 

## 4. Discussion

This study describes the real-world utilization of MOV during the Omicron surge in Israel in early 2022, specifically patient characteristics, healthcare resource utilization, and outcomes. We found that MOV-treated patients were more likely to be older and with multicomorbid conditions compared to the overall cohort of all antiviral treatment-eligible patients. Seventy-four percent were ≥65 years of age, 93.8% had ≥2 comorbidities that placed them at increased risk for severe COVID-19, and 87.7% were taking ≥1 comedication that was either contraindicated to or had major potential for drug–drug interactions with, nirmatrelvir/ritonavir. Among this group, the proportion of COVID-19-related hospitalizations and/or mortality was 3.6%, with similar rates across sexes and age groups (18–64 vs. ≥65 years) and lower rates among recently vaccinated and/or recently SARS-CoV-2-infected patients. During the study period, MOV treatment was the only option for patients who were not suitable for nirmatrelvir/ritonavir treatment due to potential drug–drug interactions or contraindications, as monoclonal antibodies were not available at that time, and encompassed 1 in 5 of every individual at MHS who was eligible for antiviral treatment.

Because the effectiveness of MOV in reducing the risk of COVID-19 hospitalizations and mortality has been established [12], this study focused on describing its real-world outcomes. It was designed as a single-arm study because of the challenges associated with defining an appropriate comparison group. Similar studies conducted in Italy [16], Japan [17], and Romania [18] have also described the real-world utilization of MOV using a single-arm design to report outcomes. The Devito et al. study [16] included a MOV-treated population that was less likely to be obese (26.6% vs. 39.6%) and slightly less likely to have had at least one COVID-19 vaccination (89.1% vs. 91.4%) compared to this one, while Kimata et al. [17] had a MOV-treated population that was slightly younger (median age: 68.0 years vs. 74.1 years) and Streinu-Cercel et al. [18] were less likely to have MOV users with cardiovascular disease (28.3% vs. 57.0%). When designing comparative effectiveness studies, the positioning between nirmatrelvir/ritonavir and MOV in COVID-19 treatment guidelines may create a channeling bias where MOV generally is prescribed to more medically complex patients, placing them at higher risk of severe COVID-19 disease. A recent feasibility study conducted during the approximate same period at MHS (1 January to 28 February 2022) aimed to assess the number of nirmatrelvir/ritonavir patients and identify appropriate control groups for a future study [29], described nirmatrelvir/ritonavir treated patients as younger than the MOV treated patients in our study (median age (IQR): 67 (59–75) years vs. 74.1 (64.3, 81.7) years), with lower levels of comorbid conditions such as chronic kidney disease (24.4% vs. 66.6%), immunosuppression (12.1% vs. 39.9%), and cardiovascular disease (22.2% vs. 57.0%). These data on nirmatrelvir/ritonavir-treated patients, together with a comparison to untreated patients [29], may be found in Supplementary Table S5. A recent study from Arbel et al. [15] assessed the effectiveness of MOV in Israel during the Omicron surge using electronic medical records from Clalit Health Services, the largest Israeli state-mandated health plan. This study reported the benefit of MOV treatment in older age groups (≥65 years and higher) in females vs. males. The subgroup results from our analysis within the MOV-treated group showed no significant differences in any outcomes based on age (although the youngest age group in our study was broader and spanned 18–64 years) or sex but did confirm differences based on vaccination status. However, efforts to make comparisons between effectiveness rates with other MOV-treated cohorts or comparison groups across studies should be made with caution due to potential differences in study design and patient characteristics.

Several limitations should be noted in this study. This was a single-arm retrospective descriptive study where treatment effectiveness was not assessed, as finding an untreated suitable control group of comparable risk with a large enough sample size to provide statistical power or adequately adjusting for differences in patient characteristics using other methods was not deemed to be feasible at study initiation. A direct comparison with the nirmatrelvir/ritonavir treated group was not possible at the time of the study due to MHS pharmacy data regulations which prevented comparisons between new treatments in the same drug class when they were first introduced. Comparison of MOV-treated patients to the group who were offered MOV but did not initiate treatment was not within the scope of this single-arm study. Nonetheless, the characteristics of MOV-treated patients are described in this study in the context of the overall cohort of antiviral treatment-eligible patients. Additional insights regarding the characteristics of antiviral treatment eligible patients who were not treated with MOV may be obtained from the feasibility study conducted at the approximate same period at MHS [29] (Supplementary Table S5). As age, sex, obesity, and overweight were not significantly associated with COVID-19-related hospitalization in univariate analyses, a multivariable analysis to evaluate the independent effect of risk factors was not pursued. Nonetheless, the univariate results presented here provide valuable data to better understand the real-life experience of MOV-treated patients by major risk factors. Except for hospitalization, HCRU was based on all-cause HCRU and could not be reliably attributed to COVID-19. Hospitalization in a COVID-19 ward was defined as COVID-19 related, though this may have been for isolation rather than the reason for hospitalization. This analysis may have missed hospitalizations outside of COVID-19 wards and those not reported by the Ministry of Health as due to COVID-19. Though MHS provides care for about a quarter of Israel’s population, the results from this study may not be generalizable to all individuals in Israel who were treated with MOV for COVID-19 during the same period. In addition, the data used in this analysis could not confirm with certainty that MOV use was started within five days of symptom onset; it was assumed based on physicians’ assessment of patient eligibility in clinical practice. Although the MHS COVID-19 antiviral treatment center was designed to deliver medication to patients’ homes within hours, information regarding the delivery time was not available for this analysis. Symptom onset and medication delivery time data could be collected from patients using questionnaires in future studies to potentially mitigate these limitations in future studies. 

Although other treatments for COVID-19 are available, including oral and intravenous antiviral agents and intravenous monoclonal antibodies, many challenges remain in ensuring that patients have access to and receive timely, appropriate, and effective therapy. All therapeutic options may not be available or accessible (e.g., due to the need for intravenous or intramuscular administration) to patients in general or within an appropriate timeframe to ensure optimization of efficacy. Additionally, some therapeutic options may not be effective due to the emergence of variants with reduced in vitro susceptibility or not be appropriate either due to drug–drug interactions or the presence of underlying medical conditions (e.g., severe hepatic or severe renal impairment), which may preclude the use of these drugs. MOV will continue to be an important and effective tool as the COVID-19 pandemic evolves with the emergence of new variants, varying degrees of vaccine uptake and effectiveness, and may, in many settings, be the only available treatment for more medically complex patients with high risk for severe COVID-19 disease.

This study population of older patients with increased risk for severe disease was observed to have a 3.3% COVID-19 hospitalization rate and a 1.1% all-cause mortality rate. While the absence of a comparison group precludes conclusions regarding treatment effectiveness, these data describe the characteristics and outcomes of patients treated with MOV for COVID-19 in Israel, whose clinical characteristics may prevent their use of nirmatrelvir/ritonavir to treat their COVID-19 infection.

In May 2023, the World Health Organization declared that COVID-19 is no longer a global health emergency. While it is impossible to predict the next pandemic, the availability of effective treatments against current and future variants is of great public health importance. Therefore, further studies are needed to explore the efficacy of MOV against new variants both in vaccinated and non-vaccinated individuals. 

## Figures and Tables

**Table 1:** Characteristics of patients in the antiviral treatment-eligible cohort (N = 5596) and MOV-treated cohort (N = 1147).

Patient Characteristics	MOV-Treated Cohort, N = 1147 ^1^	Overall Antiviral Treatment-Eligible Cohort, N = 5596 ^2^
Age, years		
Median (IQR)	74.1 (64.3, 81.7)	70.5 (61.1, 77.3)
Range	21.0, 101.0	18.6, 103.2
Age group, years		
18–64	299 (26.1%)	1947 (34.8%)
65+	848 (73.9%)	3649 (65.2%)
Sex		
Male	610 (53.2%)	2819 (50.4%)
Female	537 (46.8%)	2777 (49.6%)
Residential area		
North	219 (19.1%)	1078 (19.3%)
Sharon	231 (20.1%)	1280 (22.9%)
South	239 (20.8%)	873 (15.6%)
Center	192 (16.7%)	1095 (19.6%)
Jerusalem and Shfela	266 (23.2%)	1270 (22.7%)
Socioeconomic status		
Low	227 (19.8%)	900 (16.1%)
Med	425 (37.1%)	1910 (34.1%)
High	494 (43.1%)	2774 (49.6%)
Missing	1 (0.1%)	12 (0.2%)
COVID-19 vaccination, ever	1048 (91.4%)	4986 (89.1%)
Days since the last vaccine dose		
N vaccinated (%)	1048 (91.4%)	4986 (89.1%)
Median (IQR)	139.0 (33.0, 182.0)	151.0 (32.0, 178.0)
Previous SARS-CoV-2 infection, ever	39 (3.4%)	217 (3.9%)
Previous SARS-CoV-2 infection, last 180 days	7 (0.6%)	29 (0.5%)
COVID-19 vaccination and/or infection		
Unvaccinated (0 doses) without prior infection	89 (7.8%)	531 (9.5%)
Vaccinated (≥1 dose) and/or previously infected, with the most recent vaccine/infection >180 days prior	280 (24.4%)	1210 (21.6%)
Vaccinated (≥1 dose) and/or previously infected, with the most recent vaccine/infection ≤180 days prior	778 (67.8%)	3855 (68.9%)
BMI category (kg/m^2^)		
Underweight (<18.5)	10 (0.9%)	40 (0.7%)
Normal (18.5–24.9)	241 (21.0%)	1154 (20.6%)
Overweight (25.0–29.9)	418 (36.4%)	2078 (37.1%)
Obese (30.0+)	454 (39.6%)	2170 (38.8%)
Missing	24 (2.1%)	154 (2.8%)
Comorbidities		
Cancer	375 (32.7%)	1631 (29.1%)
Diabetes	468 (40.8%)	2130 (38.1%)
Cardiovascular disease	654 (57.0%)	2179 (38.9%)
Chronic kidney disease	764 (66.6%)	2658 (47.5%)
Hypertension	928 (80.9%)	3732 (66.7%)
Immunosuppression	458 (39.9%)	1730 (30.9%)
Liver disease	62 (5.4%)	306 (5.5%)
Neurological disorders	465 (40.5%)	2092 (37.4%)
Lung disease, any below	184 (16.0%)	739 (13.2%)
COPD	137 (11.9%)	530 (9.5%)
Asthma	43 (3.7%)	231 (4.1%)
Bronchiectasis	16 (1.4%)	60 (1.1%)
Cystic fibrosis	0 (0.0%)	1 (0.0%)
Interstitial lung disease	0 (0.0%)	2 (0.0%)
Pulmonary embolism	19 (1.7%)	32 (0.6%)
Pulmonary hypertension	3 (0.3%)	8 (0.1%)
Tuberculosis	0 (0.0%)	2 (0.0%)
Disaccharide/monosaccharide deficiencies and malabsorption	6 (0.5%)	21 (0.4%)
Sickle cell disease	0 (0.0%)	1 (0.0%)
Thalassemia	0 (0.0%)	5 (0.1%)
N comorbidities listed above ^3^		
N	1147	5596
Median (IQR)	4.0 (3.0, 5.0)	3.0 (2.0, 4.0)
Range	0.0, 8.0	0.0, 9.0
N comorbidities listed above ≥2	1076 (93.8%)	4701 (84.0%)
Smoking, ever	88 (7.7%)	484 (8.6%)
eGFR ^4^, mL/min/1.73 m^2^		
90+	175 (15.3%)	1137 (20.3%)
60–89	390 (34.0%)	2023 (36.2%)
30–59	275 (24.0%)	780 (13.9%)
15–29	43 (3.7%)	62 (1.1%)
<15	13 (1.1%)	17 (0.3%)

^1^ The MOV-treated cohort is a subset of the antiviral treatment-eligible cohort. Characteristics were summarized at MOV dispensation date; frequencies reported as n (%). ^2^ Characteristics were summarized at SARS-CoV-2 positive test date; frequencies reported as n (%). This number includes MHS members who were offered nirmatrelvir/ritonavir or MOV and were recorded to either accept or decline treatment (irrespective of treatment dispensation). ^3^ Lung diseases were grouped (counted once). ^4^ Most recent test result up to 6 months pre-index.

**Table 2:** Outcomes following MOV treatment, overall and by risk factors.

Patient Characteristics	Total N	Cumulative Incidence (95% CI), 28 Days Post-Index			
		A. COVID-19-Related Hospitalization	B. All-Cause Hospitalization	C. All-Cause Mortality	D. Composite Outcome ^1^
Overall	1147	3.3% (2.3%, 4.3%)	7.2% (5.7%, 8.7%)	1.1% (0.5%, 1.7%)	3.6% (2.5%, 4.6%)
Age group, years					
18–64	299	3.3% (1.3%, 5.4%)	5.7% (3.0%, 8.3%)	0.3% (0%, 1.0%)	3.3% (1.3%, 5.4%)
65+	848	3.3% (2.1%, 4.5%)	7.8% (6.0%, 9.6%)	1.4% (0.6%, 2.2%)	3.7% (2.4%, 4.9%)
Sex					
Male	610	3.0% (1.6%, 4.3%)	7.1% (5.0%, 9.1%)	1.6% (0.6%, 2.6%)	3.4% (2.0%, 4.9%)
Female	537	3.7% (2.1%, 5.3%)	7.4% (5.2%, 9.6%)	0.6% (0%, 1.2%)	3.7% (2.1%, 5.3%)
COVID-19 vaccination, ≥1 dose					
No	99	12% (5.5%, 18%)	18% (10%, 25%)	3.0% (0%, 6.3%)	12% (5.5%, 18%)
Yes	1048	2.5% (1.5%, 3.4%)	6.2% (4.7%, 7.7%)	1.0% (0.4%, 1.5%)	2.8% (1.8%, 3.8%)
COVID-19 vaccination and/or previous SARS-CoV-2 infection					
Unvaccinated (0 doses) without prior infection	89	12% (5.2%, 19%)	19% (11%, 27%)	3.4% (0%, 7.0%)	12% (5.2%, 19%)
Vaccinated (≥1 dose) and/or previously infected, with the most recent vaccine/infection >180 days prior	280	3.9% (1.6%, 6.2%)	7.9% (4.7%, 11%)	0.7% (0%, 1.7%)	3.9% (1.6%, 6.2%)
Vaccinated (≥1 dose) and/or previously infected, with the most recent vaccine/infection ≤180 days prior	778	2.1% (1.1%, 3.1%)	5.7% (4.0%, 7.3%)	1.0% (0.3%, 1.7%)	2.4% (1.4%, 3.5%)
BMI category (kg/m^2^)					
Normal (18.5–24.9)	241	5.4% (2.5%, 8.2%)	11% (6.8%, 15%)	1.2% (0%, 2.6%)	5.8% (2.8%, 8.7%)
Underweight (<18.5)	10	30% (0%, 53%)	30% (0%, 53%)	10.0% (0%, 27%)	30% (0%, 53%)
Overweight (25.0–29.9)	418	3.1% (1.4%, 4.8%)	7.9% (5.3%, 10%)	1.4% (0.3%, 2.6%)	3.3% (1.6%, 5.1%)
Obese (30.0+)	454	2.0% (0.7%, 3.3%)	4.6% (2.7%, 6.5%)	0.7% (0%, 1.4%)	2.2% (0.8%, 3.5%)
Missing	24	0% (0%, 0%)	0% (0%, 0%)	0% (0%, 0%)	0% (0%, 0%)

^1^ Composite outcome of COVID-19-related hospitalization and/or all-cause mortality.

## Data Availability

All data generated or analyzed during this study are included in this published article and its supplementary information files.

## Supplementary Materials

The following supporting information can be downloaded at: https://www.mdpi.com/article/10.3390/epidemiologia4030031/s1, A. Cohort definition: Table S1: Risk points contributing to the cohort definition for antiviral treatment-eligible patients. (B) Diagnosis codes for comorbidity definitions. (C) Table S2: ATC codes for medication groups. (D) Supplementary results: Table S3: ATC codes for medications contraindicated to, or with major potential for drug–drug interactions with, nirmatrelvir/ritonavir; Table S4: HCRU within 28 days post-index in the MOV-treated cohort (N = 1147); Figure S1: Outcomes following MOV treatment, by sex. Figure S2: Outcomes following MOV treatment by COVID-19 vaccination and/or infection status (E) Additional data on antiviral treatment eligible patients from another study: Table S5. Select characteristics of patients treated with nirmatrelvir/ritonavir vs. patients who were offered but did not initiate treatment from a feasibility study conducted at the approximate same period at MHS.
