# Supplementary material for: Real-World Utilization of Molnupiravir during the COVID-19 Omicron Surge in Israel
Source: Epidemiologia (Basel). 2023 Aug 10;4(3):309–21. doi: 10.3390/epidemiologia4030031 (PMC10443270; doi:10.3390/epidemiologia4030031)
Supplement: Supplementary file 1 [file epidemiologia-04-00031-s001.zip › epidemiologia-2456840-supplementary.pdf]

epidemiologia

Supplementary Material

Contents:

A. Cohort definition
- Table S1. Risk points contributing to the cohort definition for antiviral treatment-eligible patients

B. Diagnosis codes for comorbidity definitions

C. ATC codes for medication groups
- Table S2. ATC codes for medication groups

D. Supplementary results
- Table S3. Comedications contraindicated to, or with major potential for drug-drug interactions with, nirmatrelvir/ritonavir
- Table S4. HCRU within 28 days post-index in the MOV-treated cohort (N=1,147)
- Figure S1. Outcomes following MOV treatment, by sex
- Figure S2. Outcomes following MOV treatment, by COVID-19 vaccination and/or infection status

E. Additional data on antiviral treatment eligible patients from another study
- Table S5. Select characteristics of patients treated with nirmatrelvir/ritonavir vs. patients who were offered but did not initiate treatment from a feasibility study conducted at the approximate same period at MHS [29]

A. Cohort definition

Table S1. Risk points contributing to the cohort definition for antiviral treatment-eligible patients

Risk factor	Definition	Risk points
Age	*See age-specific definition of high risk**	
Obesity	Body mass index ≥ 30 kg/m^2, according to the most recent measure in the prior 5 years	1
Diabetes	Existing MHS registry[1]	1
Cardiovascular disease	Existing MHS registry[2]	1
Chronic kidney disease	Existing MHS registry[3]	1
Chronic liver disease	ICD-9 codes 570-573	1
Neurologic disease	ICD-9 codes 320-389 (≥2 on separate dates)	1
Active malignancy or malignancy treated during the past 5 years	Existing registry; MHS pharmacy data	1
Immunosuppression	Existing MHS registry (including solid organ and bone marrow transplant, asplenia, recently dispensed oncologic and non-oncologic immunosuppressive treatments, advanced chronic kidney disease and other diagnosed immunosuppression)	1
Pregnancy	Existing MHS registry	1
Recent hospitalization	Hospitalization in the past 3 years (not including delivery/birth	1 per admission
Lung disease or smoking history	Existing MHS registry for COPD; ICD-9 codes for asthma (493), bronchiectasis (494), interstitial lung disease (516.3), pulmonary embolism (515.1), pulmonary hypertension (416.0), cystic fibrosis (277.0) and lung transplant (V42.6); history of tobacco use disorder and physician reports of smoking	1
COVID-19 vaccination and prior SARS-CoV-2 positive test results	unvaccinated and never infected	3
	1 dose and never infected	2
	2 doses and >6mo since last dose	2
	3 doses and >6mo since last dose	1
	unvaccinated and infected >3mo. prior	1

*Age group was not included in the risk score, but used to define risk levels, with high risk defined as: (i) age ≥70 years; (ii) age 50-69 years with a risk score ≥2 points; (iii) age <50 years with a risk score ≥4 points. This high-risk definition was applied to patients who were deemed eligible for treatment in clinical practice by physicians in the MHS COVID-19 treatment center through February 2022.

B. Diagnosis codes for comorbidity definitions

The following ICD-9 codes were used to identify comorbidities diagnosed in the prior 12 months: Liver disease (570-573); Neurologic disease (320-389; ≥2 on separate dates); Asthma (439); Bronchiectasis (494); Cystic fibrosis (277.0); Interstitial lung disease (516.3); Pulmonary embolism (515.1); (Pulmonary hypertension (416.0); Lung transplant (V42.6); Tuberculosis (010-018); Disaccharide/monosaccharide deficiencies and malabsorption (271.3); Sickle cell disease (282.6); Thalassemia (282.4).

C. ATC codes for medication groups

Table S2. ATC codes for medications contraindicated to, or with potential for major drug-drug interactions with, nirmatrelvir/ritonavir [4-6]

Medication group	ATC codes				
Analgesic/antipyretic	N01AH01	N02AA05	N02AA08	N02AB02	N02AC04
	N02AX02	N02BB02	N02CA01	N02CA02	N02CC05
	N02CC06	N02CD04	N02CD06	N02CD07	R05DA03
Anesthetic	N01AH02	N01AH03	N01AX03	N01BB01	N01BB07
	N01BC01				
Antibiotics	D06AX01	D10AF02	J01FA09	J01FA15	J01XX12
	J04AB02	J04AB04	J04AB05	J04AK05	J04AK06
	P01AB07				
Anticonvulsant	N03AA02	N03AA03	N03AB02	N03AD01	N03AE01
	N03AF01	N03AF02	N03AG01	N03AG06	N03AX03
	N03AX22				
Antidepressant	N06AF04	N06AX05	N06AX06	N06AX18	N06AX24
	N06AX28				
Antidiabetic	A10BB01	A10BH03	A10BK02	A10BX02	A10BX03
Antiemetic	A04AA02	A04AD12			
Antifungal	J02AC02	J02AC03	J02AC04	J02AC05	J02AX07
Antihistamine	R06AX11	R06AX12	R06AX28		
Antimalarial	P01BF01	P01BX02	P01BF05	P1BF07	
Antineoplastic	L01AC01	L01CA02	L01CA05	L01CD02	L01CD04
	L01CX01	L01DB01	L01DC04	L01EB10	L01EE04
	L01EL03	L01EM04	L01EN01	L01EN02	L01EN03
	L01EX15	L01EX18	L01EX21	L01EX22	L01EX23
	L01FD03	L01FX17	L01XE03	L01XE04	L01XE06
	L01XE07	L01XE08	L01XE09	L01XE10	L01XE11
	L01XE14	L01XE15	L01XE16	L01XE17	L01XE18
	L01XE21	L01XE23	L01XE24	L01XE26	L01XE27
	L01XE28	L01XE33	L01XE38	L01XE39	L01XE42
	L01XE43	L01XE44	L01XE45	L01XE46	L01XE50
	L01XE51	L01XE53	L01XE54	L01XE56	L01XE57
	L01XX19	L01XX42	L01XX46	L01XX47	L01XX48
	L01XX52	L01XX61	L01XX62	L01XX63	L01XX69
	L01XX72	L02BA02	L02BB04	L02BB05	L02BX04
Antiparasitics	P01BA01	P01BA06	P01BC01	P01BC02	P01BX01
	P01CA03				
Antiparkinson	N04CX01	N05AX17			
Antipsychotic	N05AB10	N05AC02	N05AD01	N05AD10	N05AE04
	N05AE05	N05AF05	N05AG02	N05AH02	N05AH04
	N05AX08	N05AX11	N05AX12	N05AX14	N05AX15
	N05AX16				
Antithrombotic agents	B01AA03	B01AA04	B01AC04	B01AC23	B01AC24
	B01AC26	B01AF01	B01AF02	B01AF03	
Antivirals	J05AE04	J05AE08	J05AG02	J05AG04	J05AP05
	J05AP06	J05AP07	J05AP11	J05AP53	J05AP54
	J05AP56	J05AP57	J05AR09	J05AR10	J05AR14
	J05AR15	J05AR18	J05AR22	J05AR23	J05AR26

	J05AX09	J05AX18	V03AX03		
Cardiovascular	C01AA05	C01BA01	C01BA03	C01BB01	C01BB02
	C01BC03	C01BD01	C01BD04	C01BD07	C01EB17
	C01EB18	C02AC02	C02CA04	C02KX01	C02KX04
	C02KX05	C03BA11	C03DA04	C03DA05	C03XA01
	C03XA02	C04AE04	C05AE03	C08CA01	C08CA02
	C08CA04	C08CA05	C08CA06	C08CA07	C08CA08
	C08CA09	C08CA11	C08CA12	C08CA13	C08DA01
	C08EA02	C09XA02	C10AA01	C10AA02	C10AA05
	C10AA07	C10AX12			
Corticosteroid	A01AC01	H02AB13	H02CA02		
Dermatological	D01AC08	D07AC17	D11AH01	D11AH04	D11AX22
Gastro-intestinal	A03AE01	A03FA02	A03FA03	A06AH03	A07DA03
	A07EA06				
Genital/urinary	G02AB01	G02AB03	G02CB01	G02CX02	G03AA06
	G03AA09	G03AA10	G03AA11	G03AB05	G03AB06
	G03AB09	G03AC01	G03AC03	G03AC08	G03AC09
	G03AC10	G03FA13	G03XB01	G04BD07	G04BD08
	G04BD10	G04BD11	G04BD12	G04BE03	G04BE08
	G04BE09	G04BE10	G04BE11	G04BX14	G04CA01
	G04CA02	G04CA03	G04CA04		
Hematologic	B06AC06	B06AX04			
Hormone	H01CC03	H01CC54			
Hypnotic/sedative/anxiolytics	N05BA01	N05BA02	N05BA05	N05BA09	N05BA12
	N05BB01	N05BE01	N05CD01	N05CD03	N05CD04
	N05CD05	N05CD08	N05CF01	N05CF02	N05CF04
	N05CM19	N05CM21			
Immuno-suppressant	L04AA10	L04AA27	L04AA29	L04AA44	L04AD01
	L04AD03				
Metabolic	A16AX10	A16AX20			
Nervous system (others)	N07BB01	N07BC06	N07XX13		
Other respiratory system products	R07AX02	R07AX31	R07AX31	R07AX32	R07AX3

D. *Supplementary results*

Table S3. Comedications with a high potential for drug-drug interactions with nirmatrelvir/ritonavir

**Comedications (dispensed between 90 days pre- and 30 days post-index)**	**Antiviral treatment-eligible cohort, N = 5,596[1]**	**MOV-treated cohort, N = 1,147[2]**
Analgesic, Antipyretic	870 (15.5%)	245 (21.4%)
Anesthetic	0 (0.0%)	0 (0.0%)
Antibiotic	98 (1.8%)	26 (2.3%)
Anticonvulsant	171 (3.1%)	63 (5.5%)
Antidepressant	47 (0.8%)	14 (1.2%)
Antidiabetic	112 (2.0%)	36 (3.1%)
Antiemetic	8 (0.1%)	4 (0.3%)
Antifungal	10 (0.2%)	3 (0.3%)
Antihistamine	1 (0.0%)	0 (0.0%)
Antineoplastic	18 (0.3%)	8 (0.7%)
Antiparasitic	1 (0.0%)	0 (0.0%)
Antipsychotic	122 (2.2%)	57 (5.0%)
Antithrombotic	1,044 (18.7%)	468 (40.8%)
Antivirals	1 (0.0%)	1 (0.1%)
Cardiovascular	2,995 (53.5%)	774 (67.5%)
Costicosteroid	2 (0.0%)	1 (0.1%)
Dermatological	51 (0.9%)	10 (0.9%)
Gastrointestinal	80 (1.4%)	28 (2.4%)
Genital, Urinary	931 (16.6%)	221 (19.3%)
Hypnotic, Sedative, Anxiolytics	533 (9.5%)	177 (15.4%)
Immunosuppressants	30 (0.5%)	7 (0.6%)
Metabolic	0 (0.0%)	0 (0.0%)
N groups listed above		
≥1	4,041 (72.2%)	1,006 (87.3%)
0	1,555 (27.8%)	141 (12.3%)
1	1,936 (34.6%)	324 (28.2%)
2	1,374 (24.6%)	361 (31.5%)
3	535 (9.6%)	215 (18.7%)
4	153 (2.7%)	82 (7.1%)
5+	43 (0.8%)	24 (2.1%)

[1] Characteristics were summarized at SARS-CoV-2 positive test date; frequencies reported as n (%). This number includes MHS members who were offered nirmatrelvir/ritonavir or MOV and were recorded to either accept or decline treatment (irrespective of treatment dispensation)
[2] Subset of the antiviral treatment-eligible cohort. Characteristics were summarized at MOV dispensation date; frequencies reported as n (%).

Table S4. HCRU within 28 days post-index in the MOV-treated cohort (N=1,147)

**HCRU**	**Outcome**	**MOV-treated cohort, N = 1,147[1]**
PCP visits	Patients with ≥1 visit[1]	1,131 (98.6%)
	Patients with ≥2 visits[1]	902 (78.6%)
	Number of visits[2]	
	Median (IQR)	2.0 (2.0, 4.0)
	Range	1.0, 15.0
Specialist visits	Patients with ≥1 visit[1]	409 (35.7%)
	Patients with ≥2 visits[1]	160 (13.9%)
	Number of visits[2]	
	Median (IQR)	1.0 (1.0, 2.0)
	Range	1.0, 12.0
Telemedicine visits	Patients with ≥1 visit[1]	574 (50.0%)
	Patients with ≥2 visits[1]	253 (22.1%)
	Number of visits[2]	
	Median (IQR)	1.0 (1.0, 2.0)
	Range	1.0, 7.0
ER or after-hour urgent care	Patients with ≥1 visit[1]	108 (9.4%)
	Patients with ≥2 visits[1]	29 (2.5%)
	Number of visits[2]	
	Median (IQR)	1.0 (1.0, 2.0)
	Range	1.0, 3.0
Hospitalization (all-cause)	Patients with ≥1 admission[1]	83 (7.2%)
	Patients with ≥2 admission[1]	10 (0.9%)
	Length of stay	
	Median (IQR)	4.0 (2.0, 7.0)
	Range	1.0, 24.0
Hospitalization (all-cause) with ICU admission	Patients with ≥1 admission[1]	12 (1.0%)
	Length of stay	
	Median (IQR)	3.5 (2.0, 5.0)
	Range	1.0, 14.0
Hospitalization (COVID-19 related)	Patients with ≥1 admission[1]	38 (3.3%)
	Patients with ≥2 admission[1]	4 (0.3%)
	Length of stay	4.5 (3.0, 7.0)
	Median (IQR)	1.0, 24.0

[1] n (%) [2] Among patients with ≥1 visit.

Figure S1. Outcomes following MOV treatment, by sex

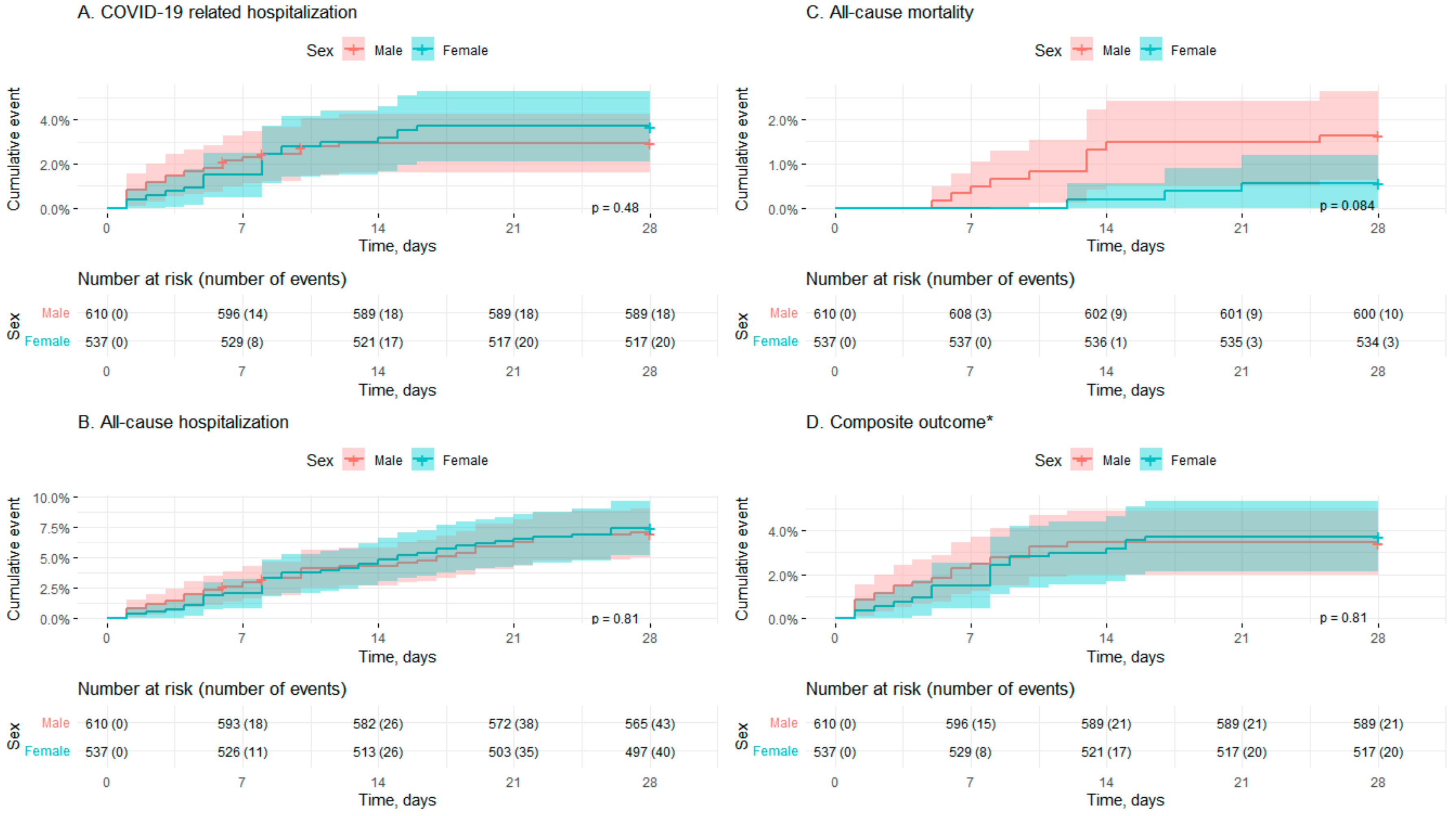

* Composite outcome of COVID-19 related hospitalization and/or all-cause mortality

Figure S2. Outcomes following MOV treatment, by COVID-19 vaccination and/or infection status

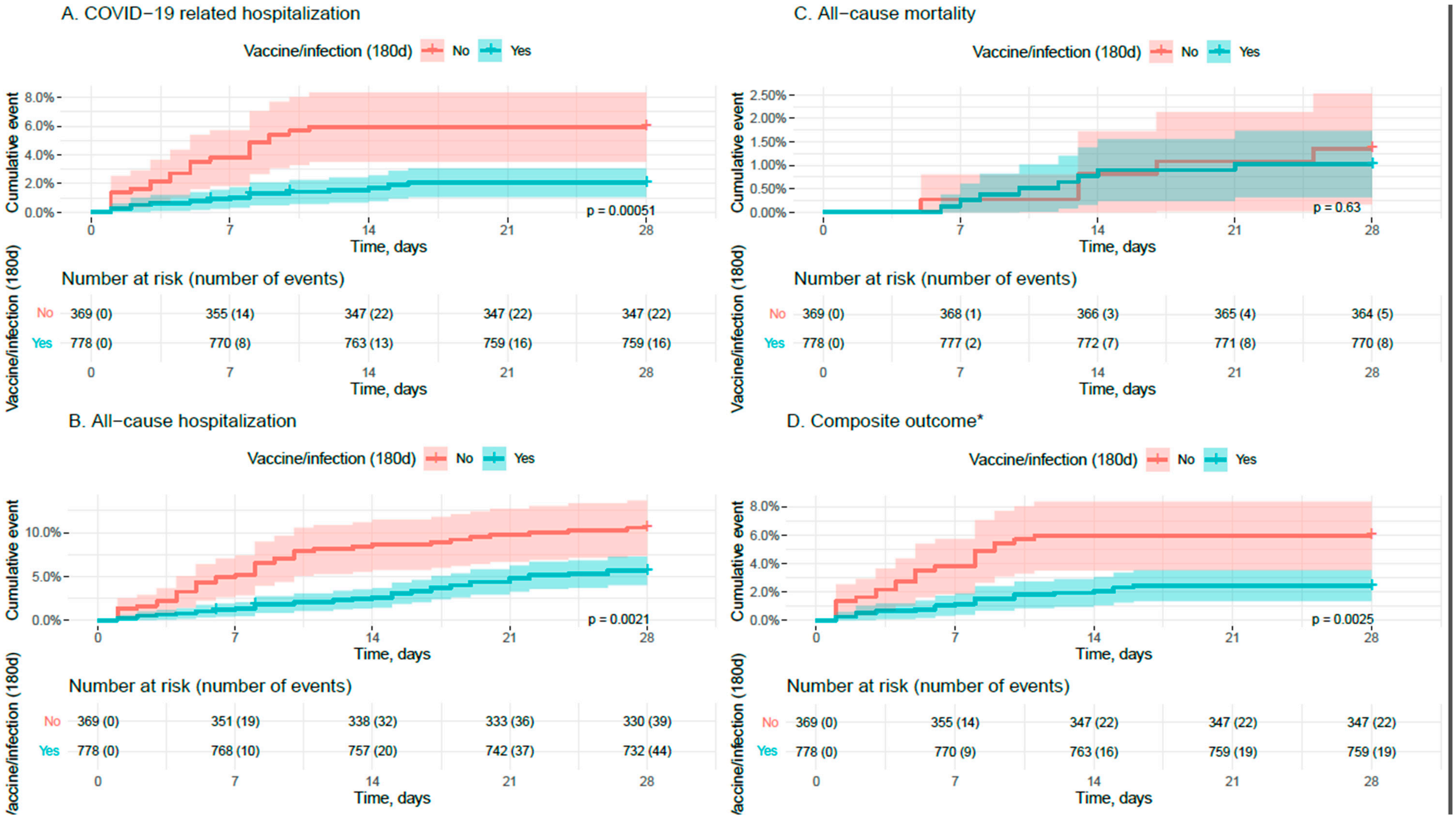

Vaccine/infection (180d) = Vaccinated (≥1 dose) and/or previously infected, with the most recent vaccine/infection ≤180 days prior.
* Composite outcome of COVID-19 related hospitalization and/or all-cause mortality

E. Additional data on antiviral treatment eligible patients from another study

Table S5. Select characteristics of patients treated with nirmatrelvir/ritonavir vs. patients who were offered but did not initiate treatment from a feasibility study conducted at the approximate same period at MHS [29]

Characteristics	Paxlovid Treated cohort (Jan 1 - Feb 28, 2022) N = 3,714		Paxlovid Untreated cohort (Jan 1 - Feb 28, 2022) N = 1,810		SD* Between Treated vs. Untreated
	N/Stat 1	%/Stat 2	N/Stat 1	%/Stat 2	SD
**Age numeric**					-0.02
Mean/Std	66.8	11.9	67.1	13.7	
N/Missing Values	3,714	0	1,810	0	
Lowest/Highest	19	103	18	100	
First/Third Quartile	59	75	59	76	
Median/IQR	67	59-75	69	59-76	
**Age group, N (%)**					0.21
≤49 years	219	5.9	182	10.1	
50-69 years	1,888	50.8	762	42.1	
≥70 years	1,607	43.3	866	47.8	
**Gender, N (%)**					-0.01
Female	1,845	49.7	902	49.8	
Male	1,869	50.3	908	50.2	
**Chronic diseases and risk conditions, N (%)**					
BMI ≥ 30 kg/m^2 (obesity)	1342	36.1	656	36.2	0.00
Diabetes mellitus	1224	33	641	35.4	-0.05
Cardiovascular disease (CVD)	826	22.2	409	22.6	-0.01
Hypertension	2158	58.1	1147	63.4	-0.11
Chronic kidney disease (CKD)	651	17.5	431	23.8	-0.16
Liver disease	908	24.4	422	23.3	0.03
Diseases of the nervous system (ICD-9)	290	7.8	137	7.6	0.00
COPD	297	8	139	7.7	0.01
Cancer (active or treated prior 5-years)	857	23.1	361	19.9	0.08
Immunosuppressed	451	12.1	227	12.5	-0.01
Hospitalization in prior 3-years	1718	46.3	851	47	-0.02

**Covid-19 vaccination and previous infection, N (%)**					
Unvaccinated with no previous infection	357	9.6	461	25.5	0.43
Unvaccinated with previous infection	6	0.2	7	0.4	
Vaccinated with no previous infection	3332	89.7	1327	73.3	
Vaccinated with previous infection	19	0.5	15	0.8	

*STD greater than .10 in absolute value suggests potentially meaningful differences.

Tene, L.; Chodick, G.; Fallach, N.; Ansari, W.; Distelman-Menachem, T.; Maor, Y. Describing COVID-19 Patients During The First Two Months of Paxlovid (Nirmatrelvir/Ritonavir) Initiation in a Large HMO in Israel. medRxiv 2022, 2022.2005. 2002.22274586.
